# Prevalence and associated factors of acute diarrhea among under-five children in Kamashi district, western Ethiopia: community-based study

**DOI:** 10.1186/s12887-020-02138-1

**Published:** 2020-05-19

**Authors:** Adugna Fenta, Kassahun Alemu, Dessie Abebaw Angaw

**Affiliations:** Department of Epidemiology and Biostatistics, Institute of Public Health, College of Medicine and Health Sciences, University of Gondar, Gondar, Ethiopia

**Keywords:** Acute diarrheal disease, Under-five children, Ethiopia

## Abstract

**Background:**

Diarrheal diseases are the second major cause of death among under-five children globally. It kills about 2.5 million people each year, with 60–70% of them being children under 5 years of age. It is also the second leading cause of morbidity in Ethiopia, with Benishangul Gumuz region bearing burden the highest with 169/1000 under five. This study aimed to determine the prevalence and associated factors of acute diarrhea among under 5 years of age children in Kamashi district, western Ethiopia, 2018.

**Method:**

A community based cross-sectional study design was used. A two-stage systematic random sampling technique was used to select 8 kebeles and 717 study units. Binary logistic regression model to identify the association between dependent and independent variables.

**Results:**

The prevalence of acute diarrhea was 14.5% (95% CI**:** (12.3, 17.3%)). Poor latrine hygiene (AOR = 11.48, 95%CI: 5.64–23.35)), had no handwashing facilities near latrines (AOR = 7.07, 95%CI:3.84–13.03), poor handwashing practice at a critical time (AOR = 5.92, 95%CI: 2.58–13.70), who stored water at home in Jerricans (AOR = 8.6, 95%CI: 1.51–48.84) and complementary feeding before 6 months (AOR = 6.49, 95%CI: 2.01–20.96) had a significant association with acute diarrhea.

**Conclusion:**

The prevalence of acute diarrhea was still high. Latrine cleanness, availability of handwashing facilities around latrine, hand washing practice at the critical time for handwashing, storage of water by “Jerrican” and time of initiation of supplementary food were the determinant factors of diarrheal diseases.

## Background

Diarrheal diseases are the second largest cause of death among children under 5 years of age globally. The World Health Organization (WHO) estimated 1.5 million children in this age group died from diarrheal diseases every year with almost half of them in Africa. The most vulnerable children are the youngest ones, particularly before their second birthday [1, 2]. Even though there is a global decline in the death rates of under-five children, the risk of a child for dying before the age of 5 years remains the highest in the WHO African Region (90 per 1000 live births), which is approximately 7 times higher than that in the WHO European Region (12 per 1000 live births) [3].

Many studies revealed that acute diarrheal disease prevalence varied from country to country. For example, in Gaza Strip, Iraq (17.7–46.1%) [4], Cameron (23.8%) [2], Tanzania (32.7%), Rwanda (26.7%) [5, 6] and in Ethiopia, ranged from 8.5 to 54% [7, 8]. In Ethiopia, the acute diarrheal diseases varied across the country: Debre Berhan referral hospital, (31.7%) [9], Arba Minch, South Ethiopia (30.5%) [10], Bahir Dar town, northwest Ethiopia (21.6%) [11], Farta district, northwest Ethiopia (16.7%) [12], Jigjiga, East Ethiopia 14.6% [13], and Adama town, Central Ethiopia, 14.7% [14] the 2016 Ethiopian Demographic Health Survey report, 12% [14], Wolaita Sodo, South Ethiopia (11%) [15] and Yeka sub-city, Addis Ababa, Central Ethiopia (8.5%) [16]. About 23% of the under 5 years child mortality in the country was caused by diarrheal diseases alone, which is greater than the annual deaths due to malaria, HIV/AIDS and measles all together [1]*.*

Exclusive breastfeeding, maternal educational status, family monthly income, source of drinking water, hand washing practice of mothers/caregivers and washing of water storage tanks had a significant association with under-five child mortality [4], quality of water used for drinking, cooking, cleaning kitchen utensils and laundry, quality of the environment in which children lived, caregivers’ knowledge of safe sources of water, type of containers used for the storage of water, age of children and child toilet facility were significantly associated with diarrhea in Sub-Saharan countries [2, 5, 6, 17]**.** In Ethiopia, inadequate water supply, lack of handwashing facilities and lack of formal maternal education [6, 18], monthly income less than birr 500 and 12–23 months of age, personal hygiene practice, food handling and storage, breastfeeding practice, and improper refuse disposal practices [3, 4, 7, 11, 12, 16, 19–23] reported as predictors of acute diarrhea among under-five children.

Although interventions, like rotavirus vaccinations, improving breastfeeding, diarrhea prevention focused on safe water and improved hygiene and sanitation were carried out, the problem remains one of the leading causes of preventable morbidity and mortality among under-five children in Ethiopia, especially in Benishangul Gumuz region.

There were few research done in a different part of Ethiopia to determine prevalence and risk factors associated with acute diarrheal diseases among under-5 year’s children at different time. But, there was a great difference in diseases burden (from 8.5 to 54%) and risk factors related to diarrhea among studies. Therefore, this study was used to determine the prevalence and associated factors with acute diarrheal diseases in Kamashi district, west Ethiopia, to develop effective prevention strategies and baseline for further research.

## Methods

### Study design, setting and period

Community-based cross-sectional study design was employed to investigate the problem in Kamashi district from August 02–16, 2018. Kamashi is one of the 20 districts in Beni Shangul Gumuz region, western Ethiopia with a projected 2018 total population of 24,572 and 3854 under five children according to Central Statistical Agency, 2007. It is located 225 Kilometer from Assosa, the capital of Beni Shangul Gumuz, and 573 km from Addis Ababa. The district has 11 health posts, a health center, one district hospital, and three private clinics.

### Sample size determination and sampling technique

**Fig. 1:**
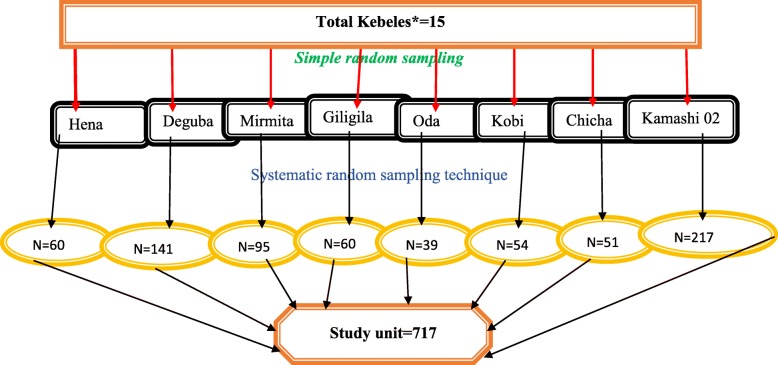
Diagrammatical presentation of proportional samples or study participants. * It is the smallest administrative unit of Ethiopia which is a part of the district.

The sample size was calculated using the single population proportion formula for the descriptive part and the double population proportion formula for associated factors. The sample size was determined by using single population proportion formula with the assumptions of 95% confidence interval (1.96), Proportion of 30.5%, design effect of two, desired precision (5%), and 10% non-response rate. The sample size for risk factor was also calculated using Epi Info version 7 with an assumption of 95% confidence interval (1.96), 80% power, ratio1:1, and outcome in unexposed. With these assumptions, the final sample size was calculated to be 717 under-five children. Because the sample selection procedure was a two-stage sampling technique, the first 8 kebeles were selected with a simple random sampling technique out of 15 kebeles. After selecting the 8 kebeles, then mothers/caregivers who had at least one child and lived in the district for 6 months was selected using the systematic random sampling technique (Fig. 1).

### Study population

All mothers/ caregivers who had at least one under-five child and had lived for at least 6 months in the district were included in the study. On the other hand, mothers or caregivers who were mentally ill, have a problem of hearing, and mothers with a critically ill child were excluded.

### Study variables, data collection procedures, and data quality assurance

Acute diarrheal disease status of under-five children is those who have three or more loose or watery stools in a 24 hours period, as reported by the mother/caretaker of the child that lasted less than 14 days was the outcome variable of the study. Independent variables like socioeconomic status (family monthly income, household size, maternal age, maternal and paternal education, ethnicity, number of under-five children, marital status, religion and age of children); environmental factors (type of water source, presence, and types of the latrine, hand-washing facility, latrine cleanness, solid and liquid waste disposal) and behavioral factors (method of water drawing and storage, feeding practices, action for diarrhea, the habit of breastfeeding, utilization of latrine, practice of waste disposal and handwashing practices at a critical time) were included in the study.

A semi-structured questionnaire was used to collect data. Ten diploma nurses who could speak both Amharic and Gumuz which is the local language were recruited as data collectors, and training was given for 2 days by the principal investigator before the actual data collection started. A pre-test was done among 5% of the sample outside the study population. Data collectors submitted the collected data on an everyday basis by incorporating their names for daily correction. Data coding and entry was checked at the beginning and midway stages of the work, while cleaning was conducted at the end of data entry.

### Data processing and analysis

After the appropriate coding, data entry was done using EPI-INFO version 7.2 and exported to SPSS version 20 for analysis. Descriptive statistics, like frequencies, percentages, and cross-tabulations were computed. Dependent and independent variables were entered into the bivariable logistic regression to detect their associations. Bi-variable analysis at a *p*-value ≤0.2 was used to select independent variables for the multivariable analysis. The backward method of variable selection was used in the multivariable analyses to identify the relative effects of explanatory variables on the dependent variable and to control confounders. Statistical significance and goodness of model fit test were checked at *p* ≤ 0.05 and the Hosmer -Lemeshow test at a *p*-value ≥0.05 respectively.

## Results

All the 717 mothers/caregivers were included in the study (100% response rate). Of the participants, 362(50%) had primary school education; 698 (97.4%) were married; 333 (46.4%) were in the age range of 24 to 29 years (mean ± SD==25.74 ± 3.92) and 346 (48.3%) of the children were 7 to 24 months of age (mean ± SD = 24.57 ± 15.17), 526 (73.4%) had one under-five child each, and 104 (14.5%) (95% CI: 12.3–17.3) reported their children had acute diarrhea 14 days before data collection (Table 1).

**Table 1:** Socio-demographic characteristics of study participant, in Kamashi district, West Ethiopia, 2018(*n* = 717)

Variables	Category	Frequency (%)
Sex	Male	400 (55.8)
	Female	317 (44.2)
Religion of caregivers/mothers	Orthodox	194 (27.1)
	Protestant	503 (70.2)
	Muslim	20 (2.7)
Educational status of mother/caregiver	Secondary	271 (37.8)
	Primary	359 (50.1)
	Illiterate	87 (12.1)
Educational status of father	Secondary	140 (19.5)
	Primary	362 (50.5)
	Illiterate	215 (30)
Ethnicity of mother/caregiver	Gumuz	431 (60.1)
	Oromo	222 (31)
	Amhara	57 (7.9)
	Others	7 (1)
Age of child in months	≤6 months	83 (11.6)
	Between 7 and 24 months	346 (48.3)
	≥25 months	288 (40.2)
Age of mother/caregiver	< 24 years	255 (35.6)
	Between 24 and 29 years	333 (46.4)
	> 29 Years	129 (18.0)
Number of < 5 years children in family	One	526 (73.4)
	Greater than one	191 (26.6)
Number of family size	≤ 5	460 (64.2)
	≥6	257 (35.8)
Health status of the child in previous 2 weeks	Had diarrhea	104 (14.5)
	No diarrhea	613 (85.5)

Of the total participants, 697(94.7%) had latrines, and 536 (74.8%) traditional pit latrines. Of these, 497(73.2%) had handwashing facilities, 647(90.2%) had no liquid waste disposal pits and 699(97.5%) used safe (public or standpipe source of water) for daily consumption (Table 2).

**Table 2:** Distribution of environmental factors of mother/caregiver in Kamashi district, West Ethiopia, 2018 (n = 717)

Variables	Category	Frequency (%)
Latrine availability (n = 717)	Yes	679 (94.7)
	No	38 (5.3)
Type of latrine (*n* = 679)	Improved	143 (19.9)
	Traditional	536 (74.8)
Frequency of latrine utilization (n = 679)	Always	649 (95.6)
	Sometimes	30 (4.4)
Cleanness of latrine (n = 679)	Yes	607 (89.4)
	No	72 (10.6)
Availability of handwashing facility in the latrine (n = 679)	Yes	497 (73.2)
	No	182 (26.8)
Availability of solid waste disposal (*n* = 717)	Yes	377 (52.6)
	No	340 (47.4)
Availability of liquid waste disposal (n = 717)	Yes	70 (9.8)
	No	647 (90.2)
Source of water for drinking purpose (n = 717)	Surface water (river)	9 (1.25)
	Public (standpipe)	699 (97.5)
	protected dug well (spring)	9 (1.25)

Of the 717 **mothers/caregiver**, 715 (99.7%) had children whose nutritional status was normal; 530(74%) had knowledge about the mode of transmission and prevention mechanisms of diarrheal diseases; 692 (96.5%) gave supplementary food to their children after 6 months, while 633(88.3%) had no knowledge about homemade treatment and its practice (Table 3).

**Table 3:** Frequency distribution of Knowledge and practice of mother/caregiver in Kamashi district, West Ethiopia, 2018(n = 717)

Variables	Category	Frequency (%)
Knowledge of diarrhea prevention and transmission mechanism	Yes	530 (74)
	No	187 (26)
Knowledge about homemade treatment for diarrhea	No	633 (88.3)
	Yes	84 (11.7)
Hand washing practice at a critical time of hand washing	Yes	319 (44.4)
	No	398 (55.6)
Starting supplementary food to the child	Before 6 months	25 (3.5)
	After 6 months	692 (96.5)
Rota vaccine status of the child	Yes	703 (98.0)
	No	14 (2)
Measles vaccination status of the child	Yes	637 (88.8)
	No	80 (11.2)
Water storage at home	“Jerrican”	709 (98.9)
	Pot	8 (1.1)

### Factors associated with acute diarrhea

After controlling all variables, the final model consisted of 5 variables which contributed to the outcome significantly with a *p*-value < 0.05. The odds of getting acute diarrhea for a child whose mother/care giver’s latrine was not clean was 11.5 times higher than that of a child whose mother/caregiver’s latrine was clean (AOR = 11.5,95% CI:5.6–23.4). Children from mother/caregivers who had no handwashing facility were 7 times more likely to get acute diarrhea compared to those with mothers/caregivers who had handwashing facilities around latrines (AOR = 7.1, 95% CI: 3.8–13.0) and for a child who started supplementary feeding before the age of 6 months, the odds of getting acute diarrhea was 6.5 times more than that of a child who started such feeding after the age 6 months (with AOR = 6.49,95% CI: 2.0–21.0) (Table 4).

**Table 4:** Multivariate analysis of diarrheal disease among under five years children in Kamashi district, Beni Shangul Gumuz region, West Ethiopia,2018(n = 717)

Variables	Category	Acute diarrhea		COR(95% CI)	AOR (95% CI)
		Yes (%)	No (%)		
Educational status of mother	Illiterate	35 (13)	236 (87)	1.12 (0.70–1.8)	0.59 (0.2–1.5)
	Primary	51 (14)	308 (86)	1.76 (0.9–3.3)	0.57 (0.2–1.4)
	Secondary	18 (20.7)	69 (79.3)	1	
Child Age Category	≤6 months	12 (14.5)	71 (85.5)	1.54 (0.98–2.42)	0.57 (0.2–1.6)
	7 to 24 months	58 (16.8)	288 (83.2)	1.01 (0.45–2.30)	
	≥25 months	34 (11.8)	254 (88.2)	1	
Family Size	≤5	74 (16)	386 (84)	1	
	≥6	30 (11.7)	227 (88.3)	1.03 (1.01–2.18)	1.13 (0.6–2.1)
Frequency of latrine utilization	Always	92 (14.2)	557 (85.8)	1	
	Sometimes	8 (26.7)	22 (73.3)	2.55 (1.08–6.00)	2.32 (0.6–8.9)
Latrine cleanness	Yes	49 (8)	558 (92)	1	
	No	51 (70.8)	21 (29.2)	27.66 (15.39–49.7)	11.48 (5.6–23.4) **
Availability of hand washing facility	Yes	26 (5)	471 (95)	1	
	No	74 (40.7)	108 (59.3)	12.41 (7.58–20.34)	7.07 (3.8–13.0) **
Presence of solid waste pit	Yes	28 (7.4)	349 (82.6)	1	
	No	76 (22.4)	264 (67.6)	3.59 (2.26–5.69)	1.78 (1.1–3.4)
Source of water (Public tab/pipe)	Protected	98 (14)	601 (86)	1	
	Unprotected	6 (33)	12 (67)	3.07 (1.13–8.36)	0.86 (0.15–4.90)
Hand washing practice at a critical time of hand washing time	Yes	31 (10)	288 (90)	1	
	No	73 (18.3)	325 (81.7)	2.30 (1.35–6.63)	5.92 (2.58–13.7) **
Initiation of supplementary food	Before 6 months	14 (56)	11 (44)	8.55 (3.75–19.23)	6.49 (2.01–20.96) **
	After 6 months	90 (13)	602 (87)	1	
Water store by jerrican	Yes	100 (14)	609 (86)	1	
	No	4 (50)	4 (50)	6.09 (1.50–25.00)	8.60 (1.51–48.84) **

Note: **significant, Hosmer and Lemeshow test *p* = 0.634 and chi-square test sig < 0.001

## Discussion

The primary aim of this study was to determine the prevalence and associated factors of acute diarrheal diseases in Kamashi district, western Ethiopia. In the current study, 104 (14.5%) of mothers/caregivers reported that their children had acute diarrhea in the previous 2 weeks. The finding was lower than those of previous studies conducted in Cameron (23.8%), Tanzania (32.7%), Senegal (26%) and Rwanda (26.7%) [2, 5, 17, 24]. That might be because of socio-cultural differences and the knowledge gap about handwashing at critical times. Mothers/caregivers who had handwashing practices at a critical time had a higher chance of interrupting feco-oral disease transmissions than those who did not wash at critical times [2, 3, 9]. In the current study, we found that knowledge about homemade treatment for diarrhea was 11.7%. This result was lower than those of studies conducted in Ethiopia like in Enderta district (54%), Debre Berhan town (31.7%), Arba Minch (30.5%), and Bahir-Dar (21.6%) [7, 9–11]. That might be because of water storage practices at home and latrine cleanness and the availability of handwashing facilities around latrines. On top of that, mothers/caregivers who stored water in narrow materials had a low probability of water contamination by hand and fetching objects. And, mothers whose latrines were clean and had handwashing facilities reduced the contamination of food and water by flies and hands [12, 16, 18].

The prevalence in this study was in line with those of studies conducted in Jig Jiga (14.6%), Serbo town (14.9%), and 2016 EDHS (12%) [3, 4, 20]. However, our result was higher than those of studies conducted in Wolaita Sodo (11%), and Yeka sub-city (8.5%) [3, 21]. The differences might be due to variations in hygiene and sanitation practices and the initiation of children into supplementary feeding**.** Children who started supplementary food before 6 months had more chance of getting acute diarrhea than those who started supplementary feeding after 6 months.

For children whose mothers/caregivers did not wash their hands at critical times, the odds of acquiring acute diarrhea were 5.9 times higher than for those whose mothers/caregivers practiced hand washing at such times. Studies in Gaza strip [4], Tanzania [17], Debre Berhan referral hospital [9], district 03, Yeka sub-city [16], and Enderta district reported similar findings [7]. This might be because mothers who did practice handwashing critical times had less chance of being infected by diarrheal disease-causing agents than mothers who did not keep their hygiene and sanitation [5, 12].

The odds of acquiring acute diarrhea for a child whose mother/caregiver did not store water in “jerricans” were 7.6 times more than those of a child whose mother/caregiver did not store water in that manner. This finding is consistent with those of studies conducted in Gaza strip [4], Tanzania [17], Debre Berhan Referral Hospital [9], Yeka sub-city [16], and Enderta district [7]. This positive association might be due to the fact that mothers/caregivers who stored water at home in wide materials had a higher probability of contaminating water by hands and objects than those who stored in narrow materials.

A child who started supplementary feeding before 6 months of age was **5.49** more likely to get acute diarrhea than a child who started supplementary feeding after 6 months. Different studies conducted in Gaza strip [4], Tanzania [17], Debrebirehan referral hospital [9], Yeka sub-city [16] Enderta district [7], Afar region [25] and systematize review in developing countries [26] reported results consistent with ours. The reason might be that a child who starts supplementary food before 6 months can be exposed to organisms that cause diarrhea and food intolerance than a child who starts after 6 months [5, 6].

For children whose mothers/caregiver’s latrines were not clean, the odds of getting acute diarrhea were 11.48 times more likely than for their counterparts. This finding is supported by those of studies conducted in Rwanda [5], Farta district [12], Geze Gofa district [6], and Kotebe sub-city [21]. The possible reason might be that there is a possibility of contacting pathogens (which causes diarrhea) in unclean environments. Therefore, mothers/caregivers whose latrine are not clean increase the probability of food and water contamination by flies and hands than those whose latrines are clean at sources and consumption.

Children whose mothers/caregivers had no handwashing facilities around latrines were 7.07 times more likely to acquire acute diarrhea compared to their counterparts. This finding is supported by those of studies conducted in Rwanda [5], Farta district [12], Geze Gofa district [6], and Kotebe sub-city [21]. Mothers/caregivers who had no hand-washing facilities around latrines had low chances of removing microorganisms that contaminant hands after latrine utilization than those who had such washing facilities [6, 22].

The limitation of the study might be the study was not engaged with seasonal differences. The prevalence of acute diarrhea reported might not reflect the actual situation that could be observed in various seasons of the year, as the information on diarrhea was collected in August, which is a wet season. It does not identify the etiological agents of diarrheal diseases because of the study design behavior.

## Conclusion

The prevalence of acute diarrhea in Kamashi district was high. Latrine cleanness, availability of handwashing facilities around latrine, hand washing practice at a critical time, storage of water by “jerrican” and time of initiation of supplementary food had a determinant factor of diarrheal disease occurrence. Therefore, health education on personal and environmental hygiene should be delivered. Additionally youth and child feeding practice should be provided to the community through a different mechanism.

## Data Availability

The datasets used and/or analyzed during the current study are available from the corresponding author on reasonable request.

## Abbreviations

AOR: Adjusted odds ratio
COR: Crude odd ratio
EDHS: Ethiopian Demographic health survey
SD: Standard deviation
WHO: World health organization
